# The Multifactorial Relationship Between Bone Tissue Water and Stiffness at the Proximal Femur

**DOI:** 10.1007/s00223-024-01327-9

**Published:** 2025-01-23

**Authors:** William Querido, No’ad Shanas, Adaeze P. Radway, Brandon C. Jones, Mikayel Ispiryan, Huaqing Zhao, Michael W. Hast, Chamith S. Rajapakse, Nancy Pleshko

**Affiliations:** 1 Department of Bioengineering, Temple University, 1947 N. 12th St, Philadelphia, PA 19122 USA; 2 Department of Biomedical Engineering, Stevens Institute of Technology, Hoboken, NJ USA; 3 Department of Radiology, University of Pennsylvania, Philadelphia, PA USA; 4 Department of Orthopaedic Surgery, University of Pennsylvania, Philadelphia, PA USA; 5 Department of Biomedical Education and Data Science, Lewis Katz School of Medicine, Temple University, Philadelphia, PA USA; 6 Departments of Mechanical and Biomedical Engineering, University of Delaware, Newark, DE USA

**Keywords:** Bone stiffness, Proximal femur mechanics, Bone tissue water, Infrared spectroscopy, Multivariate analysis

## Abstract

Bone mechanical function is determined by multiple factors, some of which are still being elucidated. Here, we present a multivariate analysis of the role of bone tissue composition in the proximal femur stiffness of cadaver bones (n = 12, age 44–93). Stiffness was assessed by testing under loading conditions simulating a sideways fall onto the hip. Compositional properties of cortical and trabecular tissues were quantified in femoral neck cross sections by Fourier transform infrared (FTIR) spectroscopy and near infrared (NIR) spectroscopy. In addition, cross-sectional areas and cortical thickness and tissue mineral density (TMD) were measured at the femoral neck. Pearson correlation analysis showed a significant (p < 0.05) negative relationship between bone stiffness and cortical and trabecular water content, both total (r = -0.63) and tightly bound to matrix and mineral (r = -55). Additionally, significant (p < 0.05) positive correlations were found between stiffness and bone area, both total (r = 0.67) and trabecular (r = 0.58). However, linear regression using each of these properties to predict bone stiffness resulted in weak models (R^2^ = 0.36–0.48). Interestingly, we found markedly stronger models (cross-validated R^2^ = 0.80–0.92) by using partial least squares (PLS) regression to predict stiffness based on combinations of bone properties. The models with highest R^2^ values were found when including bone water parameters as explanatory variables, both total and tightly bound, in cortical and trabecular. This study provides new insights by revealing a multifactorial relationship in which higher bone water content across different tissue compartments contributes to lower bone stiffness, highlighting bone water as a potential biomarker of bone quality and proximal femur mechanical function.

## Introduction

Osteoporosis is a major public health problem, prevalent in over 200 million people worldwide and rising as the aging population continuously increases [1]. The hallmark of osteoporosis is impaired bone mechanical strength, resulting in increased fracture risk associated with significant morbidity, mortality, and decreased quality of life. In particular, hip fractures are one of the most debilitating threats of osteoporosis, leading to significant levels of morbidity and more than 70% of fracture-related medical expenses in the US [1]. The most typical site of hip fractures is the proximal femur neck, where ~ 50% of all femoral fragility fractures occur [2, 3]. In routine clinical practice, bone mineral density (BMD) of the femoral neck is the primary bone property used for the diagnosis of osteoporosis and prediction of hip fracture risk [4]. Areal BMD is often assessed by dual-energy X-ray absorptiometry (DXA) in grams of mineral per square centimeter (g/cm^2^), while volumetric BMD is assessed by quantitative computed tomography (CT) in grams of mineral per cubic centimeter (g/cm^3^) [5, 6]. However, it is well recognized that assessment of BMD alone is a poor predictor of bone strength [7, 8]. Indeed, bone mechanical function has been shown to be dependent on a combination of multiple factors, including bone geometry, microarchitecture, turnover rate, porosity, tissue mineralization, collagen and mineral quality, water content, and tissue compositional heterogeneity [9–12].

For over 40 years, infrared spectroscopy has been well established as a label-free, non-ionizing tool to assess tissue composition and molecular structure [13–16]. This approach allows the simultaneously assessment of multiple components of a tissue via their “spectral fingerprint,” which comprise multiple absorbance bands that can be used for identifying and quantifying different tissue components, such as protein (primarily collagen in bone), bone mineral (biological hydroxyapatite), and water [13, 14]. There are several different modalities of infrared spectroscopy, which may be applied to investigate different types of samples and obtain specific compositional information. For example, while mid-infrared Fourier transform infrared (FTIR) spectroscopy assesses fundamental vibrations of functional groups (such as C = O, C–N, and N–H in peptides, CH_2_ in collagen, OH in water), near infrared (NIR) spectroscopy bands arise from overtones and combinations of bond vibrations [13]. FTIR is optimal to assess tissue mineral content and composition-based quality (carbonate content, HPO_4_ content, crystallinity); a great advantage of NIR is its ability to assess tissue water within a few millimeters of depth into the sample, both total and water molecules tightly bound to the matrix collagen and/or mineral [13]. These spectroscopy-based measurements of bone tissue composition have long provided great resources to investigate changes in bone properties linked to bone disease and mechanical function [10, 11, 14–20].

For example, several studies have shown that FTIR-derived spectral parameters reflecting bone mineral content, mineral properties (carbonate content, HPO_4_ content, crystallinity), and collagen maturity are altered in bone fragility diseases, such as osteoporosis and osteogenesis imperfecta (OI), as previously summarized [10, 14, 17]. In addition, Wegrzyn et al. [21] described a positive correlation between FTIR-derived collagen maturity and whole human lumbar vertebra failure load and stiffness. They also found that multivariate multiple linear regression (MLR) models combining collagen maturity to bone mass and microarchitecture improved prediction of vertebra stiffness. In a study using femoral neck samples harvested from clinical subjects undergoing hip surgery, Xia et al*.* [22] described significant correlations between femoral neck maximum load and FTIR-derived parameters (mineral content, mineral crystallinity, collagen cross-linking ratio). In a study with a mouse model of OI, Shanas et al*.* [19] found strong correlations between bone stiffness, maximum load, and post-yield displacement and NIR-determined protein content (positive correlations) and bound water content (negative correlations). In a study with human cadaveric tibiae, Hong et al*.* [20] showed that MLR analysis combining NIR-determined collagen and water parameters could better predict bone stiffness than simple regression models using either parameter alone.

Although much has been done using FTIR and NIR spectroscopy to assess the relationship between bone tissue composition and mechanics, there is currently a lack of studies into combining multiple spectroscopy-derived compositional parameters to predict bone mechanical function at the proximal femur neck [23–25], which, as described above, is the most typical site of hip fractures [2, 3]. This represents a critical gap in knowledge, as further understanding of the combined role of underlying factors that contribute to femoral neck mechanics is necessary to elucidate hip fracture mechanisms and to inform future studies on strategies to improve prediction of hip fracture risk and therapeutic management. In a recent study, Jones et al*.* used MLR analysis to show that combining BMD with bone porosity and/or cortical thickness significantly improved assessment of proximal femur stiffness [26]. Using cadaveric femurs, they showed that the R^2^ of models predicting mechanically determined stiffness increased from 0.34 when using BMD as the only explanatory variable to 0.57–0.78 in models using bone porosity and/or cortical thickness as co-variates, with or without BMD [26]. Here, we aim to further the understanding of the multifactorial tissue properties underlying proximal femur stiffness by focusing on the role of diverse bone compositional properties at the femoral neck, including bone tissue-level water, while also considering cross-sectional areas and cortical thickness and tissue mineral density (TMD).

**Fig. 1:**
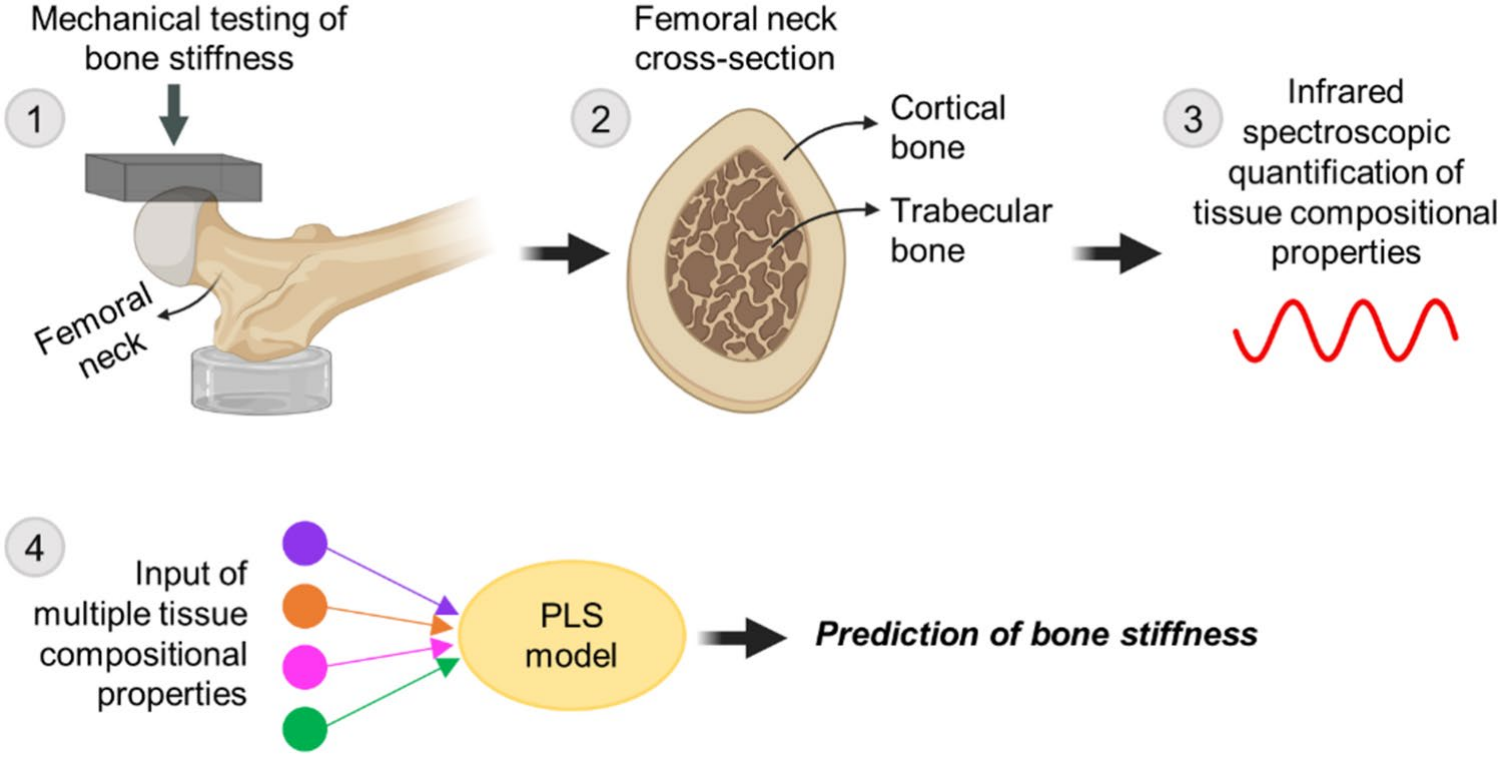
Study design and main steps

In this study, we assessed cortical and trabecular tissue composition in cadaveric femoral neck sections by using both FTIR and NIR spectroscopy to obtain a comprehensive assessment. Proximal femur stiffnesses were measured using whole bones under loading conditions that reflect a sideways fall onto the hip, considering that 90% of hip fractures are the result of a fall [1]. For data analysis, we created regression models to predict mechanically determined proximal femur stiffness, using both simple regressions based on individual predictor variables and multivariate partial least squares (PLS) regression using combinations of properties as predictor co-variables [27–29]. The study design and main steps are illustrated in Fig. 1. We hypothesized that (1) prediction of bone stiffness based on the combination of multiple bone tissue properties will be stronger than models using individual properties as single explanatory variables, and (2) parameters describing bone water will play a major role in models assessing proximal femur bone stiffness.

## Materials and Methods

### Cadaveric Bones

Whole femurs from twelve donors (nine males, four females; aged 44–93) were obtained from the National Disease Research Interchange (NDRI, Philadelphia, PA). All tissues were screened for infectious diseases, fresh-frozen at -80 °C, shipped in dry ice, and stored frozen until analysis. Freeze–thaw cycles were kept to a minimum, and the same number of cycles were used for each analysis.

## Assessment of Cortical Tissue Mineral Density

Cortical volumetric TMD at the femoral neck of intact femora were obtained by clinical computed tomography (CT) using a Revolution HD scanner (GE Medical Systems) and 50, 100, and 300 g/cc calcium rod calibration phantoms (Multi-Energy CT Phantom; Sun Nuclear Corporation), which were placed next to the bones within the field of view during the scans. Scans were performed using an established clinical axial bone imaging protocol with the following parameters: 120 kVp, tube current 150 mAs, rotation time 0.8 s, bone convolution kernel, 0.3 to 0.4 mm in-plane voxel size, 1.25 mm slice thickness, 0.5 mm slice increment, field of view 205 × 205 × 500 mm, and matrix size 512 × 512 × 1000. The calibration curve between Hounsfield Units (HU) and vTMD was determined by taking 7 equally spaced manually selected regions of interest (ROI) covering the entire length of each calibration phantom and performing a 3-point fit to the known concentrations of the calcium rods. The fit was found to be stable between all scans (R^2^ > 0.99), and hence a single calibration equation was used for all scans: TMD (mg/cc) = 0.3286 × HU – 14.95. An elliptical ROI was drawn in the compact cortical region in the inferior neck. Segmentation was performed in the Mimics 17.0 software.

## Assessment of Proximal Femur Stiffness

Following previously published protocols [20, 26, 30, 31], femora were held rigidly in place with the greater trochanter in contact with the ground and the femoral head in contact with a polymethylmethacrylate (PMMA) cup designed to imitate the acetabulum. A 3-degree of freedom vise firmly held the femoral shaft roughly 10° above the horizontal to model the contact orientation of a sideways fall onto the hip [32]. Loads were applied with a servo-hydraulic universal test frame (8874, Instron, Inc.) using a 25 kN load cell. All femurs were subjected to three triangular waveforms of non-destructive compressive loading force between -50 N and -1200 N at a loading rate of 1.67 mm/s. Time (sec), displacement (mm), and force (N) were recorded at 100 Hz throughout the test. Bone stiffness (N/mm) was quantified as the slope of the linear (elastic) region of the last load–displacement curve. Femora were not tested to failure so that the bones were preserved for subsequent analysis with spectroscopy.

## Assessment of Bone Tissue Composition

**Fig. 2:**
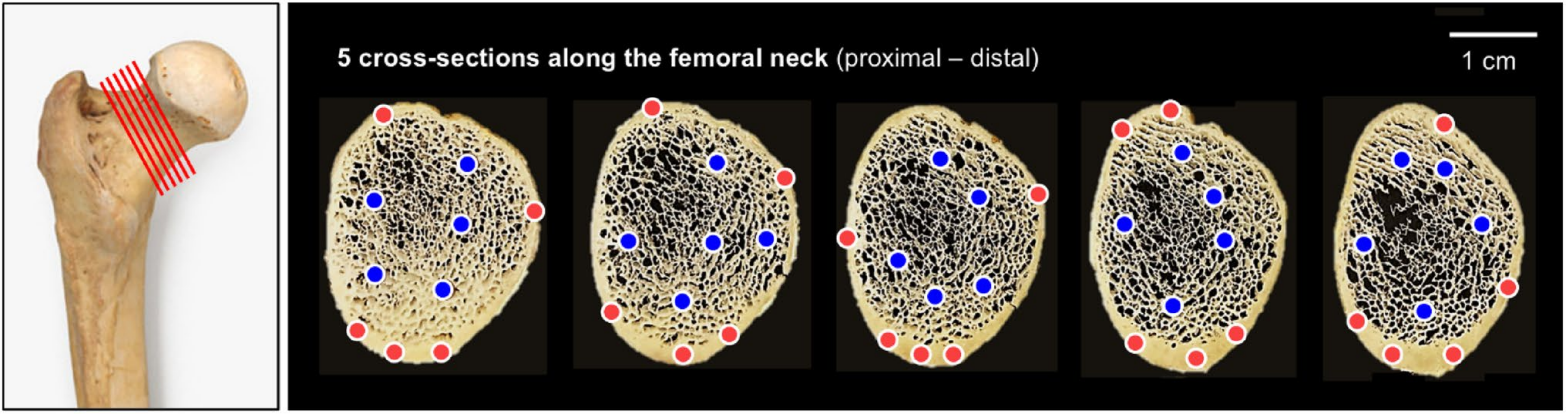
Representative images of five cross sections of the same femur depicting cortical (red) and trabecular (blue) regions (~ 2–3 mm spots) from which FTIR and NIR spectra were collected. Depending on the sample, spectra were collected from 3–5 unique sites per section

Following mechanical testing, proximal femora were cut with a hand saw and the femoral neck was sectioned with a bone saw (IsoMet 1000 Precision Cutter). A sequence of five 1-mm-thick sections was cut along the femoral neck, then cleaned to remove marrow and fat using 1% tergazyme solution under sonication at 37 °C for 60 min (3 washes of 20 min each) [33]. Regions of cortical and trabecular bone were analyzed using FTIR and NIR spectroscopy as previously described [13, 33, 34]. For FTIR, sections were lyophilized and analyzed using a Nicolet iS5 FTIR spectrometer coupled to an iD7 attenuated total reflection (ATR) accessory with a ~ 2-mm-diameter diamond crystal (Thermo Scientific). The tissue surface was placed in close contact with the crystal, and spectra were collected with 8 cm^−1^ resolution and 32 co-added scans in the 4000–400 cm^−1^ frequency range. For NIR, sections were analyzed both hydrated (pat dry with absorbent wipes to remove surface water) and lyophilized using a LabSpec 4 visible/NIR spectrometer with a low-OH fiber optic probe with a ~ 3 mm diameter (ASD). The probe tip was placed at a 2 mm distance from the tissue surface and spectra were collected with 1 nm resolution and 50 co-added scans in the 10,000–4000 cm^−1^ frequency range. For both FTIR and NIR, three to five unique spectra were acquired from cortical and trabecular bone in each of the five sections cut per bone, summing to 15–30 measured regions per bone for each method. Representative images depicting regions from which FTIR and NIR spectra were collected are shown in Fig. 2.

Spectra were analyzed using the Unscrambler software (CAMO), applying standard Savitzky–Golay smoothing and second derivative filters as a mathematically objective approach to resolve and quantify spectral peaks. To plot positive peaks, spectra were inverted (multiplication by -1). Analysis of frequency positions, contour, and intensities of the second derivative peaks were used to inform on the presence, molecular environments, and amount of tissue components [13, 14]. FTIR outcomes were mineral content (relative to protein) (1010/1660 cm^−1^ ratio), CO_3_ content (relative to mineral) (870/1010 cm^−1^ ratio), HPO_4_ content (relative to mineral) (1105/1010 cm^−1^ ratio), and mineral crystallinity (955/1105 cm^−1^ ratio). NIR outcomes were total water content (relative to protein) (hydrated samples; 5300/5800 cm^−1^), and water content tightly bound to matrix collagen and/or mineral (relative to protein) (lyophilized samples; 5300/5800 cm^−1^ ratio). For each femur, an average of each FTIR and NIR outcome was obtained from the 15–30 spectra acquired from cortical or trabecular tissues.

## Assessment of Bone Areas and Cortical Thickness

Bone areas and cortical thickness of femoral neck cross sections were obtained by image analysis using ImageJ, applying the measure tool and the slice geometry tool of the BoneJ plugin for bone image analysis. As outcomes, total bone area (mm^2^), cortical bone area (mm^2^), trabecular bone area (mm^2^), and cortical thickness (mm) were measured.

## Statistical Analysis

Paired T-tests were used to compare compositional properties between femoral neck cortical and trabecular bones. Analyses were carried out with PRISM software (GraphPad); data passed the normality test and differences were considered significant at* P* < 0.05. The degree of correlation between individual tissue properties and stiffness was expressed as the Pearson’s correlation coefficient (*r*); significant correlations were considered at *P* < 0.05. All regression analyses were done using explanatory variables standardized to Z-score values (mean = 0, SD = 1) to facilitate comparison of coefficients. Simple linear regression was carried out using JMP (SAS Institute); significant models were considered at *P* < 0.05 derived from the F-test. Multivariate regression analysis was carried out by PLS regression applying leave-one-out cross-validation using The Unscrambler (CAMO). Although PLS regression is not affected by multicollinearity among the predictors [27–29], we checked the correlation between co-variates, which were not significant, as well as their variance inflation factor (VIF), which were < 5, indicating the absence of multicollinearity issues. Models were considered significant when all predictor variable coefficients were significant contributors to the model, individually presenting *P* < 0.05. PLS outcomes were the cross-validated *R*^2^ (CVR^2^) and root mean squared error of cross-validation normalized by the mean (nRMSECV).

## Results

### Assessment of Bone Tissue Properties

**Fig. 3:**
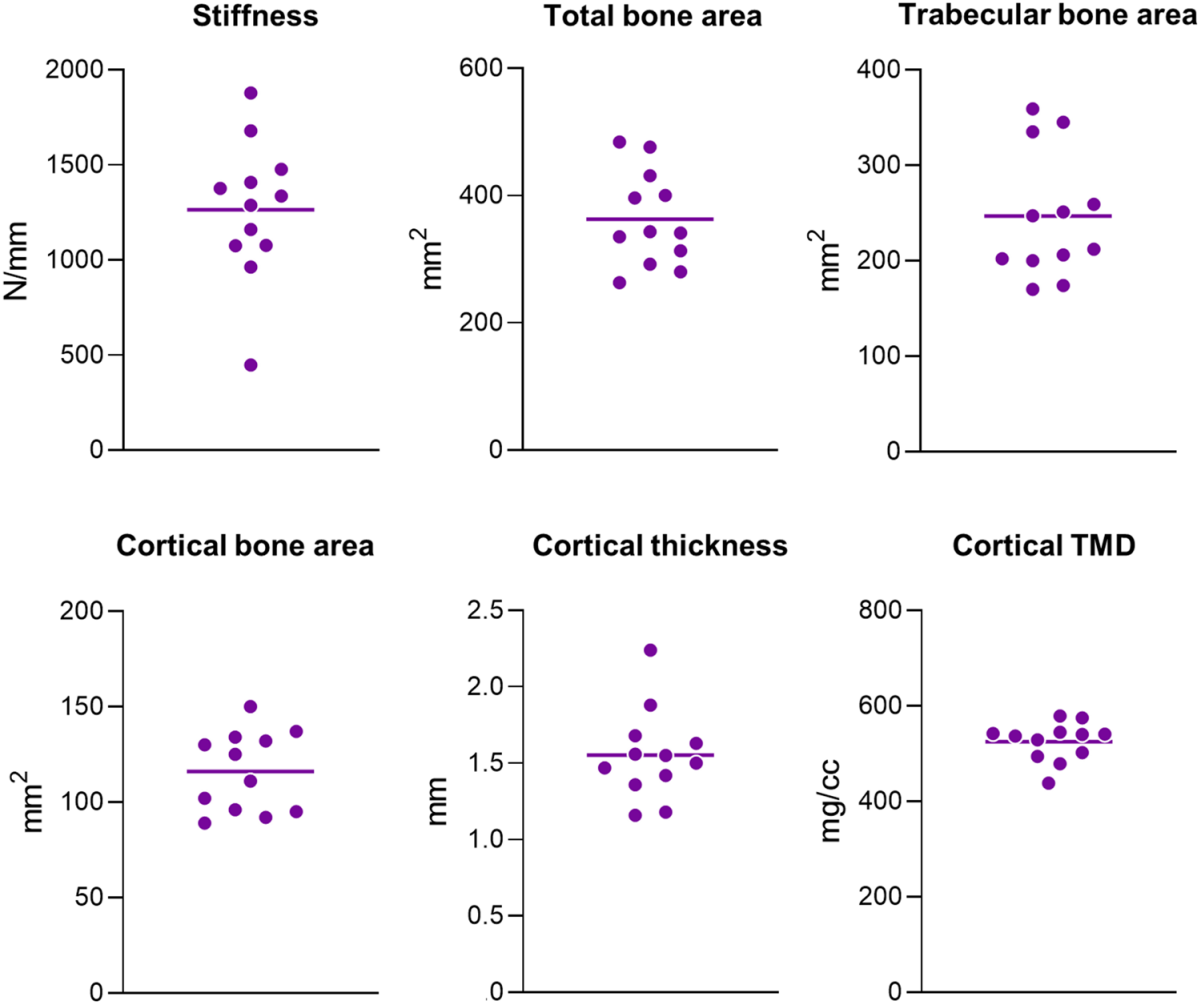
Range of proximal femur stiffness and femoral neck cross-sectional areas and cortical thickness and tissue mineral density (TMD) (*N* = 12). There were no significant outliers (Grubbs’ test)

**Fig. 4:**
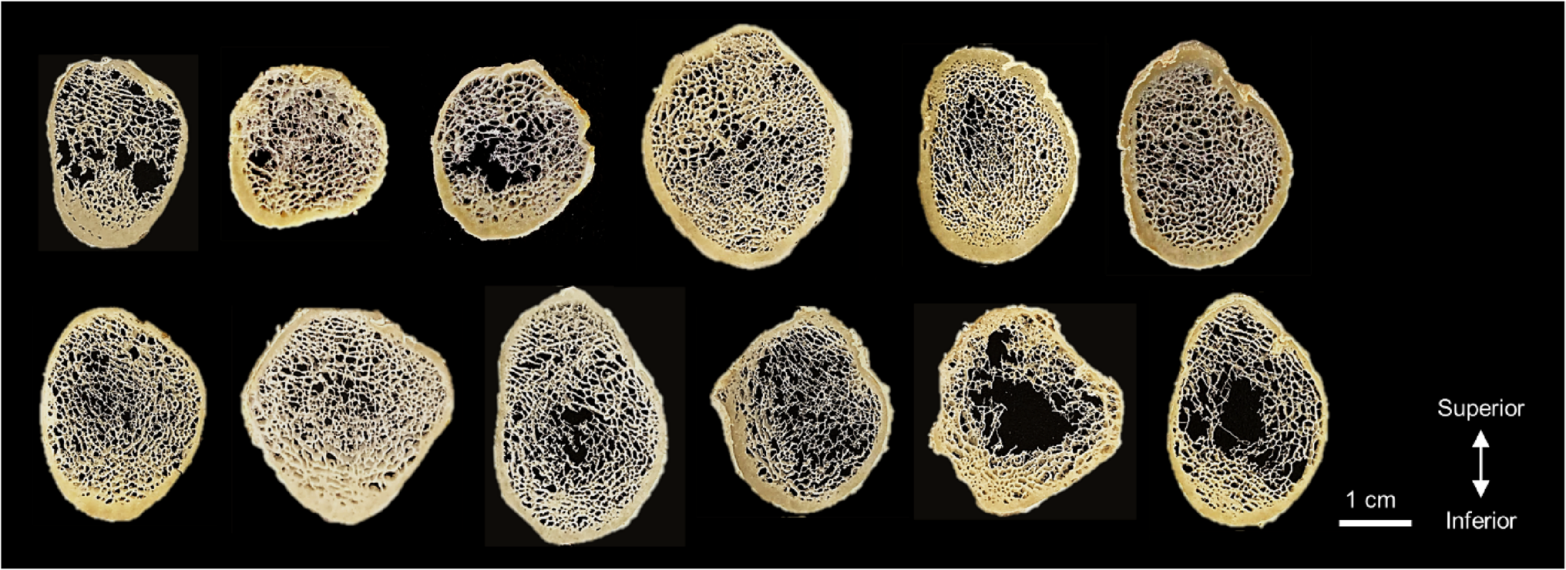
Femoral neck cross sections from different donors (*N* = 12)

**Fig. 5:**
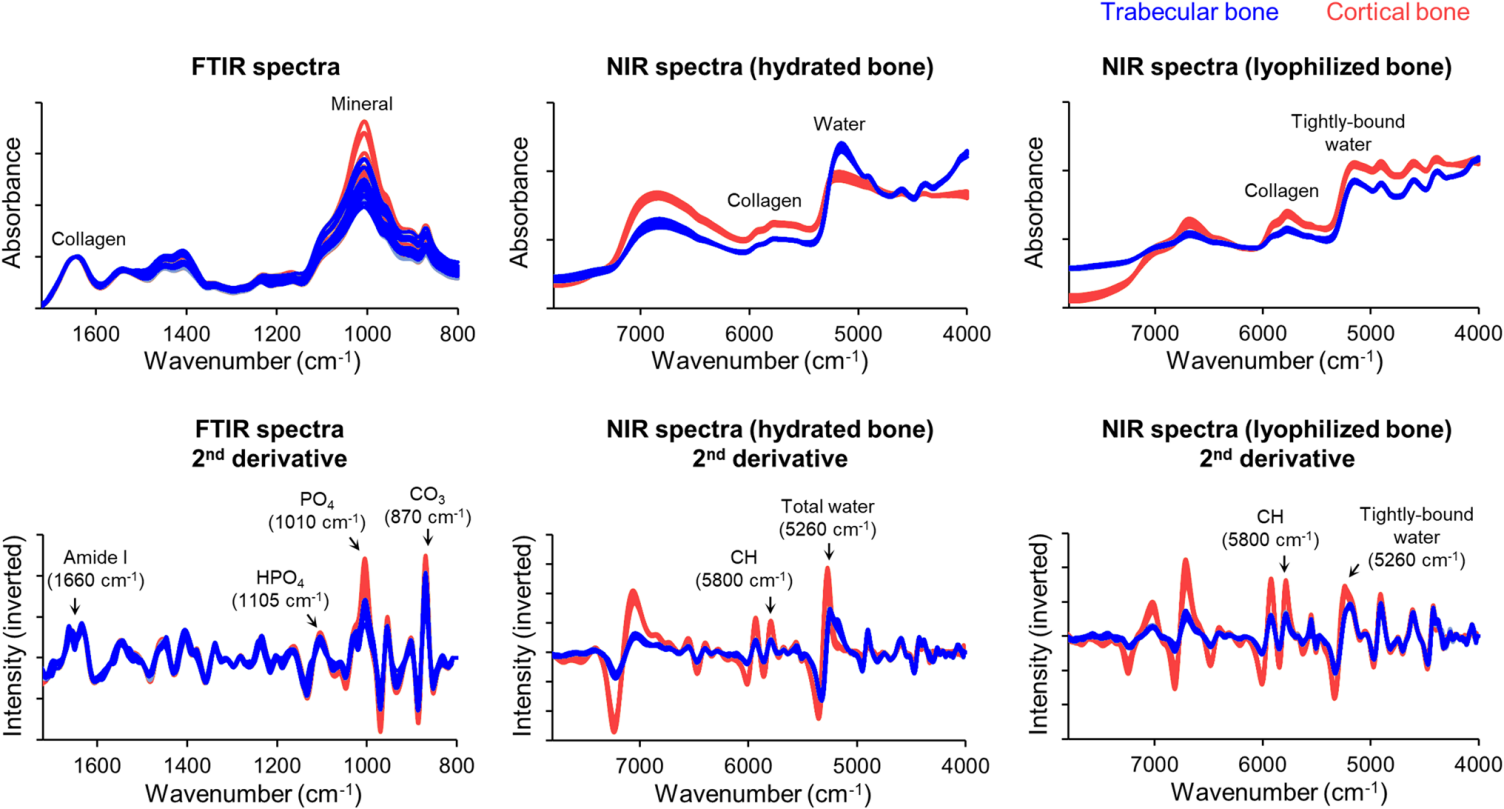
Average spectra collected using FTIR and NIR spectroscopy from bone tissues at the femoral neck (trabecular and cortical bone) (*N* = 12). Spectral labels identify peaks associated with typical bone tissue components

**Fig. 6:**
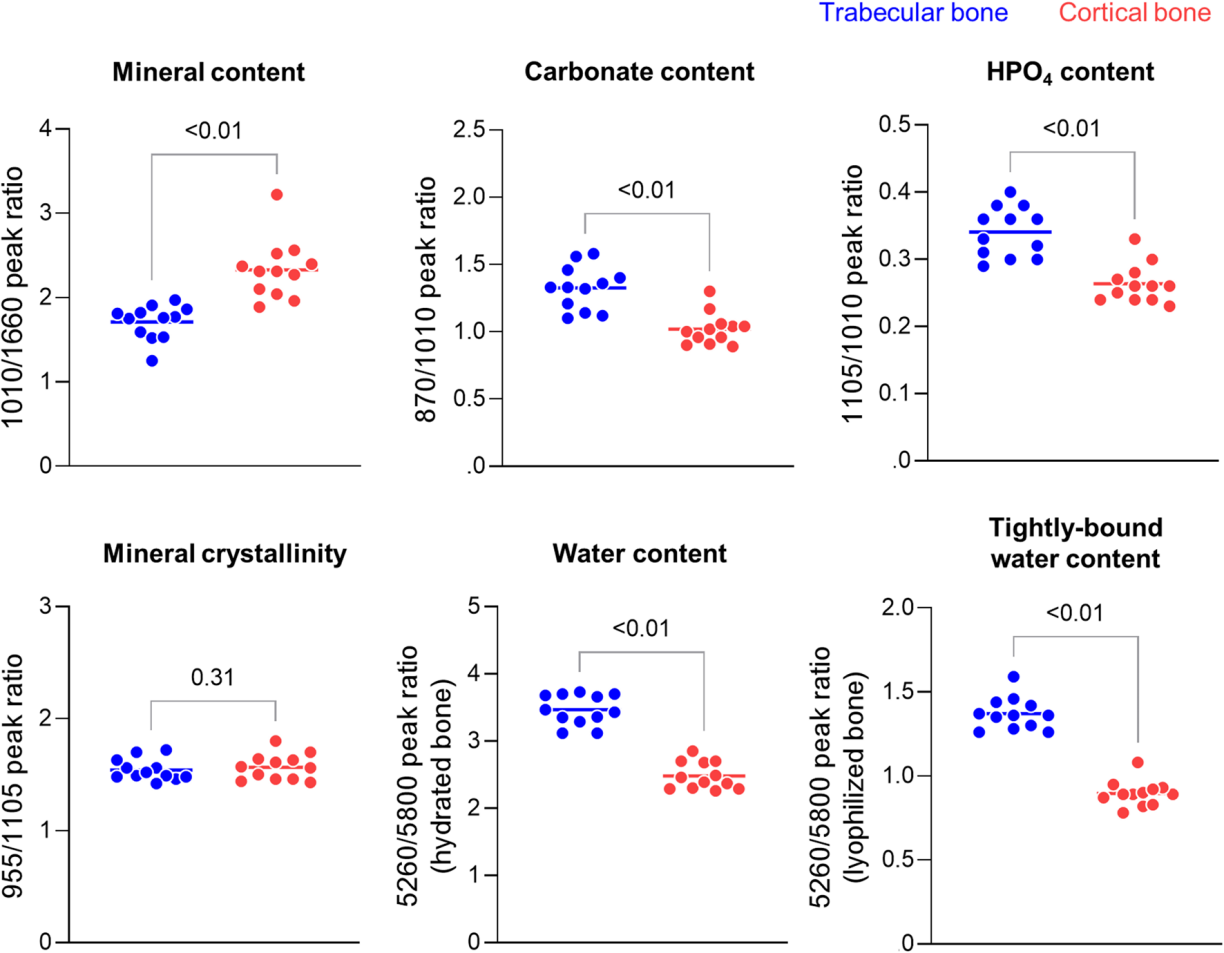
Quantification of tissue-level bone compositional properties at the femoral neck (trabecular and cortical bone) (*N* = 12). Y-axis labels show quantified spectral parameters based on 2nd derivative peak ratio

There was a range of stiffness and cross-sectional areas and cortical thickness and TMD values among the donors (Fig. 3). This diversity can be appreciated by visualizing images of femoral neck cross sections from each donor (Fig. 4). For assessing bone tissue composition, FTIR and NIR spectroscopic of trabecular and cortical tissues allowed identification (Fig. 5) and quantification of typical bone components (Fig. 6). Of highlight, compared to trabecular bone, cortical bone had a significantly greater mineral content relative to protein, with its mineral component presenting lower content of carbonate and HPO_4_ substitutions, indicative of a more mature bone mineralization. Mineral crystallinity, reflecting structural order and size of the apatite nanocrystals, was not different in cortical and trabecular bone. Moreover, cortical bone showed a lower total water content and tightly bound water content than trabecular bone, suggesting lower amount of water molecules free (pore water) and bound to matrix components (collagen, mineral).

## Correlations of Tissue Properties with Bone Stiffness

Among bone tissue compositional properties, only water content parameters showed significant negative correlations with bone stiffness, in both trabecular and cortical tissues (Table 1). There were also significant correlations between cross-sectional areas and bone stiffness (Table 1). These results indicate that lower water content and greater bone area were associated with greater bone stiffnesses.

**Table 1:** Correlations between different bone parameters and proximal femur stiffness (N = 12)

	Trabecular bone		Cortical bone	
	r	P	r	P
Mineral content	0.27	0.34	0.01	0.97
Carbonate content	0.01	0.99	0.07	0.81
HPO_4_ content	− 0.04	0.89	0.03	0.91
Crystallinity	0.23	0.44	0.11	0.70
Total water content	− **0.64**	0.01	− **0.63**	0.02
Tightly bound water content	− **0.55**	0.04	− **0.55**	0.04

	r		P	
Total bone area	**0.67**		0.01	
Trabecular bone area	**0.58**		0.03	
Cortical bone area	0.51		0.07	
Cortical thickness	-0.06		0.83	
Cortical TMD	0.54		0.07	

Bold r-values highlights significant correlations (P < 0.05)

## Multivariate Regression Analysis to Assess Bone Stiffness

Simple linear regression using individual tissue properties to predict bone stiffness showed poor model outcomes (Table 2). Interestingly, markedly stronger multivariate PLS models were obtained by combining multiple tissue properties as explanatory variables to predict proximal femur stiffness (Table 3). Of highlight, cortical and trabecular bone water content parameters were recurrent in all models, and the strongest model was obtained by combining cortical and trabecular tissue water properties without properties related to bone area or cortical thickness or TMD.

**Table 2:** Simple linear regression using individual bone properties to assess proximal femur stiffness (*N* = 12)

	R^2^	P	nRMSE
Total water content of trabecular bone	0.44	0.01	19%
Total water content of cortical bone	0.40	0.02	20%
Tightly bound water content of trabecular bone	0.38	0.02	20%
Tightly bound water content of cortical bone	0.30	0.04	21%
Total bone area	0.46	0.01	20%
Trabecular bone area	0.36	0.04	21%

nRMSE: Root mean square error (RMSE) normalized by the stiffness range

**Table 3:** Multivariate PLS regression combining different bone properties as explanatory variables to predict proximal femur stiffness (N = 12)

	CVR^2^	nRMSECV	Coefficient (Standardized)	Predictor P-value
Total water content of cortical bone	0.92	8%	-193	< 0.01
Total water content of trabecular bone			-98	0.03
Tightly bound water content of trabecular bone			-218	< 0.01
Total water content of cortical bone	0.87	10%	-247	< 0.01
Tightly bound water content of trabecular bone			-242	< 0.01
Cortical thickness	0.87	10%	84	0.04
Total water content of cortical bone			-277	< 0.01
Tightly bound water content of trabecular bone			-228	< 0.01
Cortical TMD	0.80	12%	127	0.04
Total water content of trabecular bone			-214	< 0.01
Tightly bound water content of trabecular bone			-142	0.03

Cross-validated *R*^2^ (CVR^2^) is presented as the typical metric to assess the predictive power of the PLS models. nRMSE: Root mean square error of cross validation (RMSECV) normalized by the stiffness range

## Discussion

There is a great demand for the development of approaches to improve assessment of bone strength and associated hip fracture risk. Here, we present a multivariate analysis that highlights the potential contribution of combining bone tissue water parameters to predict proximal femur stiffness. The importance of bone composition and geometry to bone mechanics is well established [9–12]. This study is novel because of its multivariate approach, which reveals the combined role of different bone properties related to the ultimate mechanical function of the proximal femur, including different types of tissue water content in both cortical and trabecular bones. In this study, we showed that multivariate regression models to predict bone stiffness based on the combination of multiple tissue properties were markedly stronger than models using individual properties as single explanatory variables. This outcome sheds new insights to advance our understanding of the multifactorial role of tissue properties in proximal femur stiffness, with emphasis on the role of cortical and trabecular bone water, beyond the role of any individual properties alone.

We used PLS leave-one-out cross validation to create and validate the multivariate models. This machine learning technique is reliable and widely used to evaluate model fitting and predictive ability by performing a *k*-fold cross-validation in which the number of *k* subsets is equal to the number of observations in the dataset [27–29]. In other words, it creates *k*–1 iterative models (training sets) in which one data point (testing sets) is excluded at a time and used as input to validate its independent prediction by the model created without it. In comparison with MLR, PLS offers advantages such as suitability for models with small sample sizes and numerous explanatory variables, and its ability to handle multicollinearity among co-variates without compromising model performance [27–29]. In addition, whereas MLR relies on adjusted *R*^*2*^ as a metric to evaluate the goodness-of-fit of models while accounting for the number of predictor variables, PLS typically utilizes the cross-validated *R*^*2*^ (also expressed as *Q*^*2*^) [27–29]. Cross-validated* R*^*2*^ is preferred in PLS because it not only reflects the fit of the model to the training data but also provides a measure of predictive accuracy and robustness when applied to unseen data, thus mitigating the risk of overfitting that adjusted *R*^*2*^ cannot detect [35]. Outcomes from this approach showed that the combination of cortical and trabecular total and tightly bound water was a significant contributor to markedly improved models, with water content being inversely correlated with bone stiffness. This suggests that tissue water content may be an important potential biomarker of bone quality.

This result is in line with several previous studies that have shown an association between tissue water content and bone mechanics in both clinical [36–38] and pre-clinical studies [19, 39]. Here, our findings expand the current knowledge by assessing water content in both cortical and trabecular tissues of the femoral neck, a region prone to fragility fractures and thus of great clinical relevance [2]. To help interpreting the findings of this study, we need to delve into how water can be found in bone tissue [33, 40, 41]. Water comprises around 20% of bone tissue in volume, being distributed in different compartments throughout the tissue. Free or pore water is located within bone porosity at the microscale, such as in the Haversian canals and the lacuno-canalicular system; loosely bound water is associate with the surfaces and interfaces between collagen fibrils and mineral crystals at the submicron and nanoscales; and tightly bound water is found as part of the collagen triple helical structure or the mineral lattice at the molecular and atomic scales. Loss of bone water by dehydration has been shown in many studies to lead to an increase in stiffness [41], which is in accordance with our overall results.

Interestingly, it is possible to delve deeper into this inverse relationship between bone water and stiffness considering water content in different tissue compartments. Free water content is a direct surrogate of cortical bone porosity [20, 42], such that a greater free water content directly reflects a greater bone porosity, which has been linked to a decrease in bone stiffness [40]. Loosely bound water plays key roles in transferring loads by promoting sliding between collagen and mineral; a reduction in this interface water content impairs this process, resulting in stiffer bones [40, 43]. Tightly bound water is thought to provide structure to the length of collagen fibrils [38, 44] and order and mechanical stability to mineral–mineral interfaces via their surface hydrated layers [45, 46]; a loss of tightly bound water may lead to a disruption in the mineralized matrix at the molecular and crystal level, leading to stiffer and more brittle bones [47].

Here, using NIR spectroscopy, we describe water content in two different bone tissue compartments: total water (which includes free and loosely bound water) and tightly bound water associated with collagen and mineral [33], assessed both in cortical and trabecular tissues. Our results not only corroborate previous findings, but also suggest that assessment of water content in different tissue compartments may provide a valuable approach to improve prediction of bone mechanical function. This is illustrated, for example, when a multivariate PLS model using as input the combination of total water content of both cortical and trabecular bone and tightly bound water content of trabecular bone more thoroughly explains whole-bone stiffness (i.e., leads to a higher *R*^*2*^ value) than models based on analysis of water on an individual tissue compartment. Overall, this shows that to elucidate how bone water assessment may inform on bone mechanical function, multiple water pools within bone tissues should be taken into consideration.

In standard clinical practice, BMD scores are often combined with other clinical risk factors (such as female sex, older age, low body mass index) via the predictive algorithm FRAX Fracture Risk Assessment Tool to predict 10-year fracture risk in individuals aged 50 years and older [8, 48]. However, the sensitivity of FRAX to predict fracture risk remains low at ~ 50%, missing nearly half of the women at high risk of hip fractures [7, 8]. Here, our study is motivated by the need to improve fracture risk prediction; however, current applications of infrared spectroscopy for a comprehensive assessment of bone composition are not suitable for in vivo evaluations. Thus, further consideration is necessary to discuss a translational pathway in which tissue compositional properties, especially bone water content measurements, may be implemented in clinical systems to predict fracture risk. Bone water is particularly interesting because it can be assessed in clinical settings by ultra-short echo time (UTE) magnetic resonance imaging (MRI) [20, 37, 42], with MRI outcomes correlated to those obtained by NIR spectroscopy [20, 42]. Specifically, UTE-MRI can be used clinically to assess free water, loosely bound water, and total water in bone, while further MRI-based approaches are being explored to assess tightly bound or structural water [40]. Moreover, with advances of new spectroscopic methods using fiber optic probes, including Raman and NIR spectroscopy, clinical assessment of bone tissue composition and quality may be on the horizon [39, 40, 49, 50]. Ultimately, the translational potential of this study is on laying a foundation for future studies aiming to combine and incorporate different types of bone water content metrics to improve multivariate algorithms in clinical models for predicting hip fracture risk.

It is also important to discuss other limitations of this study. Although the small sample size available (N = 12) is not optimal for robust statistical modeling, our results reveal a promising and novel direction towards the better understanding and assessment of bone mechanical function at the proximal femur. This may lay a foundation for future studies with larger samples sizes and different cohorts of donors, focusing for example on postmenopausal women at higher risk of typical osteoporotic femoral neck fractures [51]. Another limitation of this study is that we used stiffness instead of ultimate load as the parameter for correlation of bone biomechanics. Stiffness was chosen because it can be obtained without loading the bone to failure, allowing to preserve the intact femoral neck for structural and compositional analyses after mechanical testing. Stiffness is often used as a surrogate for bone strength due to the significant correlation between these parameters [52–56]; however, in order to directly investigate the relationship between tissue properties and bone strength, future studies may consider assessing the ultimate load instead of stiffness.

This study provides new insights into the complex relationship between bone tissue water content and bone stiffness. By investigating total and tightly bound water across cortical and trabecular tissues from femoral neck samples, we found that higher bone water content was consistently linked to lower proximal femur stiffness. Importantly, our multivariate models, which incorporated multiple tissue properties as explanatory variables, revealed a synergistic effect: the combination of water content across different tissue compartments resulted in better predictions of stiffness than any individual metrics alone. These findings suggest that bone water may serve as a valuable biomarker for assessing bone quality, offering new opportunities to better understand bone fragility and fracture risk. Additionally, this research sets the stage for future clinical and pre-clinical studies, highlighting the potential for incorporating bone water measurements into advanced diagnostic tools and in the design of tissue-engineered materials optimized for bone mechanical performance.
